# A systematic review of the sensitivity and specificity of lateral flow devices in the detection of SARS-CoV-2

**DOI:** 10.1186/s12879-021-06528-3

**Published:** 2021-08-18

**Authors:** Dylan A. Mistry, Jenny Y. Wang, Mika-Erik Moeser, Thomas Starkey, Lennard Y. W. Lee

**Affiliations:** 1 Oxford University Hospitals, Headley Way, Headington, Oxford, OX3 9DU UK; 2 University of Oxford, Oxford, UK; 3 Institute of Cancer and Genomic Sciences, University of Birmingham, Edgbaston, Birmingham, B15 2TT UK

**Keywords:** Coronavirus, COVID-19, SARS-CoV-2, Lateral flow device, Lateral flow test, Viral antigen detection, Rapid antigen detection, Reverse transcriptase polymerase chain reaction, Mass testing, Population testing

## Abstract

**Background:**

Lateral flow devices (LFDs) are viral antigen tests for the detection of SARS-CoV-2 that produce a rapid result, are inexpensive and easy to operate. They have been advocated for use by the World Health Organisation to help control outbreaks and break the chain of transmission of COVID-19 infections. There are now several studies assessing their accuracy but as yet no systematic review. Our aims were to assess the sensitivity and specificity of LFDs in a systematic review and summarise the sensitivity and specificity of these tests.

**Methods:**

A targeted search of Pubmed and Medxriv, using PRISMA principles, was conducted identifying clinical studies assessing the sensitivity and specificity of LFDs as their primary outcome compared to reverse transcriptase polymerase chain reaction (RT-PCR) for the detection of SARS-CoV-2. Based on extracted data sensitivity and specificity was calculated for each study. Data was pooled based on manufacturer of LFD and split based on operator (self-swab or by trained professional) and sensitivity and specificity data were calculated.

**Results:**

Twenty-four papers were identified involving over 26,000 test results. Sensitivity from individual studies ranged from 37.7% (95% CI 30.6–45.5) to 99.2% (95% CI 95.5–99.9) and specificity from 92.4% (95% CI 87.5–95.5) to 100.0% (95% CI 99.7–100.0). Operation of the test by a trained professional or by the test subject with self-swabbing produced comparable results.

**Conclusions:**

This systematic review identified that the performance of lateral flow devices is heterogeneous and dependent on the manufacturer. Some perform with high specificity but a great range of sensitivities were shown (38.32–99.19%). Test performance does not appear dependent on the operator. Potentially, LFDs could support the scaling up of mass testing to aid track and trace methodology and break the chain of transmission of COVID-19 with the additional benefit of providing individuals with the results in a much shorter time frame.

**Supplementary Information:**

The online version contains supplementary material available at 10.1186/s12879-021-06528-3.

## Background

Lateral flow device (LFD) immunoassays are common, inexpensive, readily available testing devices that are used in the detection of a number of different medical conditions [1–4]. They work by binding of conjugated antibodies to a specific antigen in a sample. This antibody-antigen complex moves via capillary flow to a test area which then identifies a positive test by the presence of a coloured line [2, 3].

There has been an increasing number of papers reporting on the use of LFDs in the detection of the Severe Acute Respiratory Syndrome Coronavirus 2 (SARS-CoV-2), which has caused the Coronavirus disease 2019 (COVID-19) pandemic [5]. Currently, the gold standard for detection of SARS-CoV-2 is reverse transcriptase polymerase chain reaction (RT-PCR) [6, 7]. For both of these tests, nasopharyngeal swabs are used to isolate the antigen. However, RT-PCR requires swabs to be sent off to a laboratory with specialist equipment and analysed by trained laboratory staff. This usually has a turnaround time that is variable but of at least 24 h [1, 7]. Furthermore, many countries possess a limited capacity to perform RT-PCR tests, hindering their ability to engage in mass-testing with RT-PCR alone; as an example, the United Kingdom’s current RT-PCR capacity for the detection of SARS-CoV-2 is approximately 500,000 tests per day [8].

Where there are national or local outbreaks, it is important to be able to expand testing in a short time frame (surge-testing) to enable effective identification of individuals infected with the virus for contact tracing and mass population testing in an endeavour to stop the chain of transmission of the virus [5, 9]. Lateral flow devices (LFDs) offer a potential solution as they can quickly turn around a result in less than 30 min without the need for specialist staff or laboratory capacity [2, 3]. Many countries have pioneered the use of LFDs for surge-testing in the healthcare, community and educational setting [10, 11].

To date, there has yet to be a systematic review to assess the sensitivity and specificity of LFDs in the detection of SARS-CoV-2 without which a thorough evaluation of the efficacy of these tests cannot be undertaken.

The primary objective was to identify the sensitivities and specificities of lateral flow devices in the detection of SARS-CoV-2 compared to reverse transcriptase polymerase chain reaction in patients with symptoms of COVID-19 or those screened as part of mass testing programmes. This study also set out to identify if there were any differences in sensitivity and specificity between different manufacturers of LFDs and between different operators of the LFD test.

## Methods

### Study design

This was a systematic review of clinical studies in peer reviewed journal articles.

### Search strategy

Two independent reviewers conducted an electronic search strategy of two online databases, PubMed and Medxriv, in 1st December 2020 to 15th January 2021. Search terms used included but not exclusively a combination of “COVID-19”, “SARS-CoV-2”, “CORONAVIRUS”, “ANTIGEN DETECTION”, “ANTIGEN TEST”, “LATERAL FLOW”. The two reviewers then reviewed each paper generated from the search and excluded articles based firstly on title then abstract and then reviewing the full text. References of the filtered papers were searched for additional studies. Any disagreements between the reviewers were resolved by consulting a separate adjudicator and a discussion between all three parties.

### Eligibility and exclusion criteria

Eligible studies had to meet the following criteria: (1) involved the detection of SARS-CoV-2, (2) the intervention was a LFD detecting the antigen to this virus, (3) the LFD was performed at the point of care on samples taken for this purpose, (4) the control used as the “gold standard” must be RT-PCR, (5) outcomes for the paper must include the sensitivity and specificity of the lateral flow device, (6) population must be adults (≥ 18 years) who displayed symptoms of COVID-19 or swabbed as part of screening or mass testing, (7) the full text must be published in peer reviewed journals or a preprint pending review at the time of the search.

Exclusion criteria included any study that did not meet all the conditions for eligibility and: (1) was detecting anything other than SARS-CoV-2, (2) retrospectively tested samples which had been frozen, (3) tested exclusively healthy volunteers with no indication for swabbing, (4) did not provide appropriate sensitivity and specificity data.

### Data extraction

Once all papers from the search had been identified the two independent reviewers reviewed the full text of all identified papers. Descriptive data for each article were identified including author, month and year, location, sample size and manufacturer of LFD used. The reviewers then extracted test result data including the number of participants in which SARS-CoV-2 was detected by RT-PCR and LFD and the number of false positive and negative results detected by LFDs. Sensitivity and specificity data were collected for each study including 95% confidence intervals; in all studies, this was calculated to confirm the sensitivity and specificity data. The data was subsequently split and pooled based on the manufacturer of LFD used which enabled calculation of sensitivity and specificity for each manufacturer of LFD compared to RT-PCR. Studies were split again if the sample was taken by a trained professional or if it was taken by the patient with self-swabbing, regardless of who operated the LFD test. Sensitivity and specificity data were calculated comparing these two groups. Again, any disagreements during data extraction were settled by consulting the third party.

### Outcomes

The pre-defined primary outcome was to assess the sensitivity and specificity of LFD tests in the detection of SARS-CoV-2 compared to RT-PCR (“gold standard”) testing in patients with symptoms consistent with COVID-19 or in individuals swabbed as part of mass population testing/contact tracing. The secondary outcome was to calculate the sensitivity and specificity of each LFD test by manufacturer in this same population in comparison to RT-PCR and based upon whether the sample collection was performed by a trained professional or by the patient (“self-swabbing”).

### Data analysis

Data analysis was conducted using IBM SPSS Version 27.0.0. For the primary outcome in the majority of studies, no data analysis was required as all results were extracted from articles directly. For the secondary outcome, results of individual manufacturers of LFDs were pooled together and a sensitivity/specificity analysis conducted. A total sensitivity and specificity were reported for each manufacturer with 95% confidence intervals. Data visualisation was performed in R version 4.0.3. Heatmaps and Forest plots were generated using the pheatmap() function of the ‘pheatmap’ (v1.0.12) and forestplot() function of the ‘forestplot’ (v1.10.1) R packages, respectively. Bar plots, horizontal dot plots and pie charts were generated using the geom_bar(), geom_line(), geom_point() and coord_polar() functions of the ‘ggplot2’ (v3.3.2) R package, respectively.

## Results

**Fig. 1:**
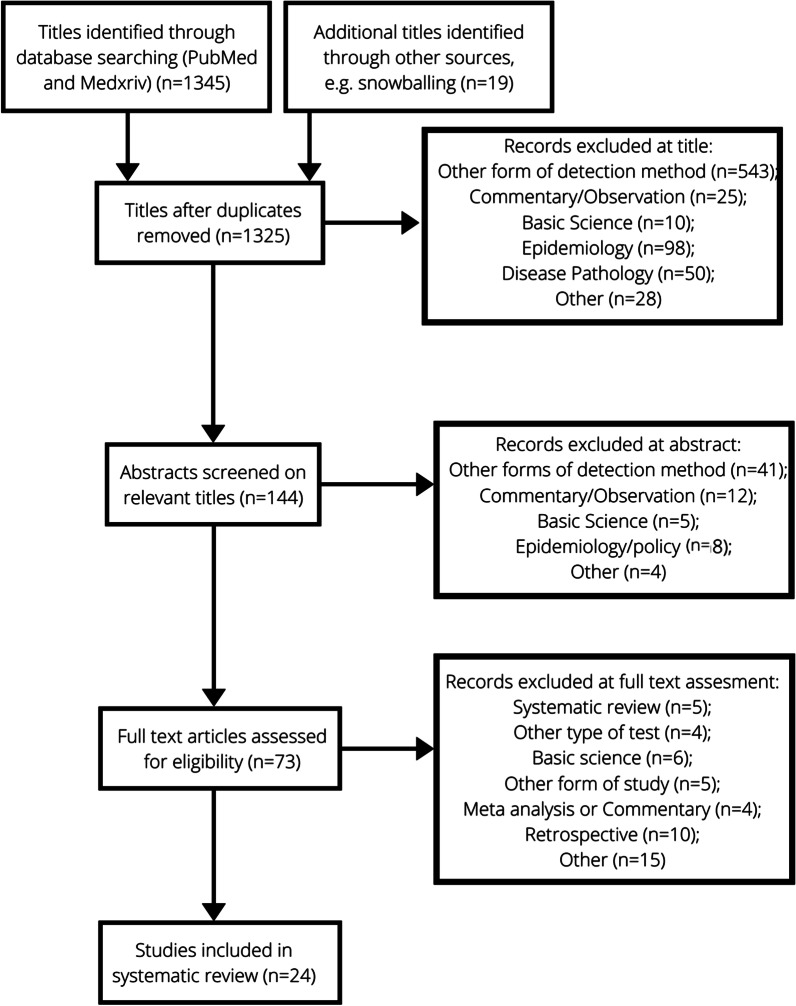
PRISMA flowchart showing systematic processing of articles

**Fig. 2:**
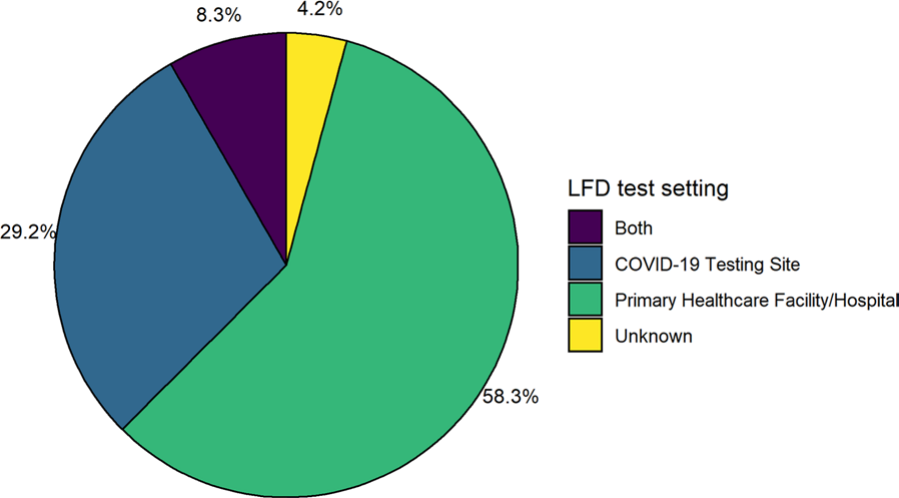
The different test setting between the studies—includes a variety of test centres and primary care centres

The search strategy yielded 1345 papers and further titles were identified by checking the references of these articles. This was narrowed down to 24 full text articles as demonstrated by the PRISMA flow diagram from in Fig. 1. In total 26,903 tests were included in these 24 articles, which are summarised in Table 1, including sample sizes, population and LFD type used. There was an almost equal gender split and a range of different test centres such as COVID-19 test centres and primary care centres (Fig. 2 and Additional file 1: Appendix 1).

**Table 1:** Data describing study design, population and setting

Study	Month and year of publication	Origin	Sample size	Gender = Female	Gender = Male	Mean Age	Population	Setting—Dichotomise	Sample collection (who collected it and when)	Intervention (which LFD)
Abdelrazik et al. [12]	December 2020	Egypt	310	126	184	42.0	Confirmed, contacts and exposed healthcare professionals	Primary Healthcare Facility/Hospital	N/A	BIOCREDIT
Abdulrahman et al. [13]	December 2020	Bahrain	4183	1820	2363	30.9	Mildly symptomatic	COVID-19 Testing Site	Trained healthcare professionals	Panbio
Albert et al. [14]	November 2020	Spain	412	239	173	31.0	Symptomatic	Primary Healthcare Facility/Hospital	Trained healthcare professionals	Panbio
Berger et al. [15]	November 2020	Geneva, Switzerland	529	285	244	34.9	Symptoms/contact	COVID-19 Testing Site	Trained healthcare professionals	Panbio; STANDARD Q
Blairon et al. [16]	August 2020	Belgium	774	N/A	N/A	N/A	N/A	Primary Healthcare Facility/Hospital	N/A	Coris
Bulilete et al. [17]	November 2020	Mallorca, Spain	1369	743	626	42.5	Symptoms/contact	COVID-19 Testing Site	Trained healthcare professionals	Panbio
Cerutti et al. [18]	September 2020	Italy	330	134	196	44.6	Symptomatic/high-risk travel	N/A	N/A	STANDARD Q
Chaimayo et al. [19]	November 2020	Thailand	454	231	223	40.4	Symptomatic	Primary Healthcare Facility/Hospital	N/A	STANDARD Q
Courtellemont et al. [20]	October 2020	France	248	131	117	43.0	Asymptomatic and symptomatic	Primary Healthcare Facility/Hospital	Trained personnel	COVID-VIRO
Drevinek et al. [21]	November 2020	Czech Republic	591	327	246	40.0	Symptoms/contact	Primary Healthcare Facility/Hospital	N.A	Panbio; STANDARD Q
Gremmels et al. [22]	October 2020	Netherlands	1575	844	523	36.4	Symptomatic	Primary Healthcare Facility/Hospital	N/A	Panbio
Iglὁi et al. [23]	November 2020	Rotterdam, Netherlands	970	776	194	53.0	Symptomatic	COVID-19 Testing Site	Trained personnel	STANDARD Q
L.J. Krüger et al. (2020) [24]	December 2020	Heidelberg and Berlin, Germany	1108	78	1030	39.4	Symptoms/contact	COVID-19 Testing Site	Trained personnel	Panbio
L.J. Krüger et al. (2020) [25]	October 2020	Berlin and Heidelberg, Germany. Liverpool, UK	2417	1276	1140	40.4	Symptoms/contact	Both	N/A	Bioeasy, Coris, STANDARD Q
Linares et al. [26]	October 2020	Madrid, Spain	255	148	107	46.4	Symptoms/contact (ER), both asymptomatic and symptomatic (72.1%) in PH	Primary Healthcare Facility/Hospital	N/A	Panbio
Masiá et al. [27]	November 2020	Alicante, Spain	913	490	423	40.6	Symptoms/contact	Primary Healthcare Facility/Hospital	Trained healthcare professionals	Panbio
Merino-Amador et al. [28]	November 2020	Madrid and Basque Country, Spain	958	587	370	42.4	Symptoms/contact	Primary Healthcare Facility/Hospital	Trained healthcare professionals	Panbio
Moeren et al. [29]	October 2020	Netherlands	352	N/A	N/A	N/A	Symptomatic	COVID-19 Testing Site	Trained personnel	BD Veritor
Nalumansi et al. [30]	October 2020	Uganda	262	29	233	34.0	N/A	Primary Healthcare Facility/Hospital	Laboratory personnel	STANDARD Q
Peto et al. [31]	January 2021	Multiple sites across the UK	6954	N/A	N/A	N/A	RT-PCR-confirmed diagnosis of SARS-CoV-2 infection within 5 days of the original PCR result	Both	Self-test	Innova
Porte et al. [32]	October 2020	Santiago, Chile	127	59	68	38.0	Symptoms/contact	Primary Healthcare Facility/Hospital	Trained personnel	Bioeasy
Schwob et al. [33]	November 2020	Lausanne, Switzerland	928	455	473	31.0	symptomatic	COVID-19 Testing Site	NP = health professional, saliva = self	STANDARD Q; Panbio; COVID-VIRO
Torres et al. [34]	December 2020	Spain	634	355	279	37.0	Asymptomatic contacts	Primary Healthcare Facility/Hospital	Trained healthcare professionals	Panbio
Veyrenche et al. [35]	September 2020	Montpellier, France	65	N/A	N/A	N/A	Asymptomatic and symptomatic	Primary Healthcare Facility/Hospital	N/A	Coris

**Fig. 3:**
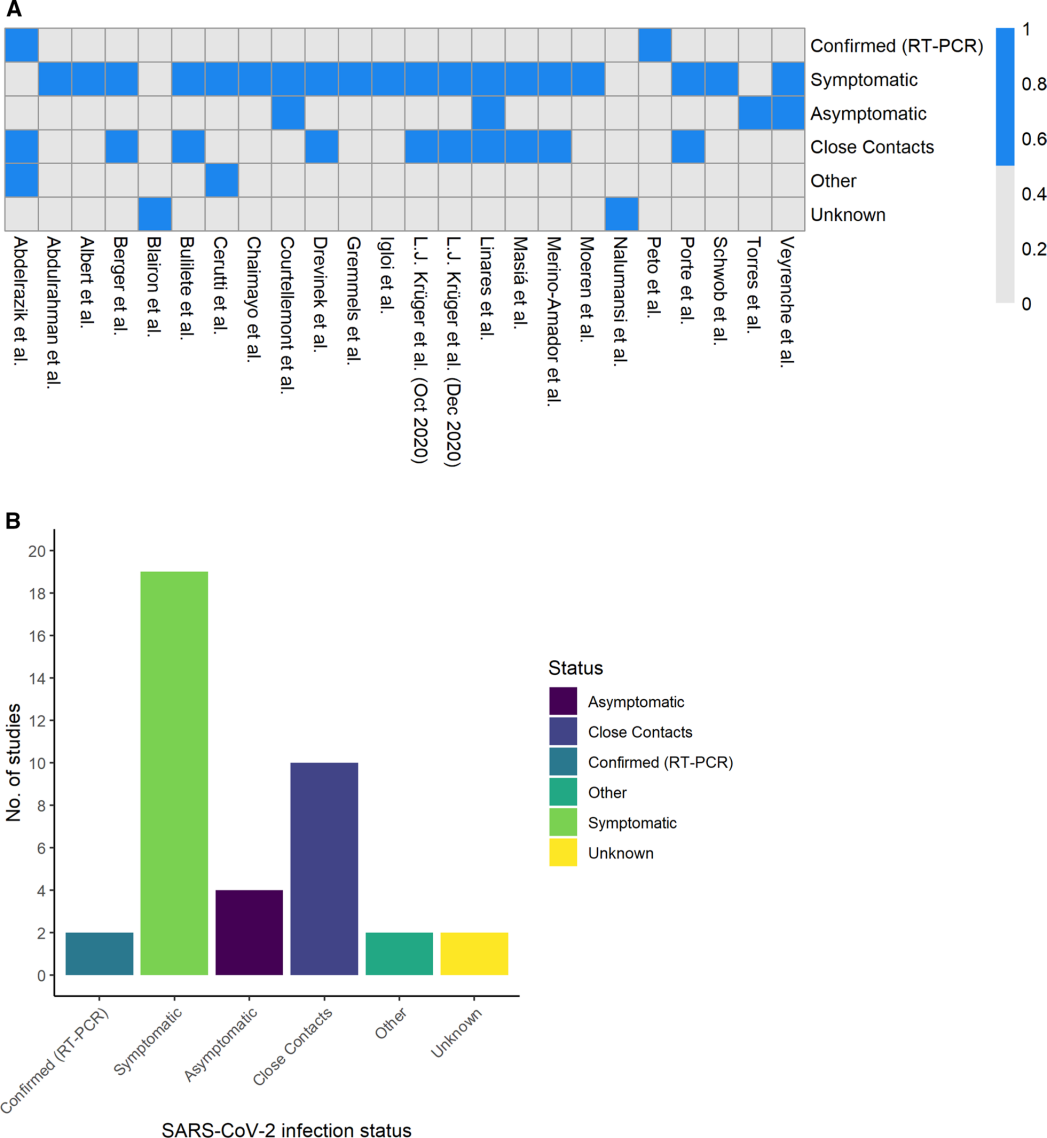
SARS-CoV-2 infection status shown across each individual paper in the heat map chart (**A**) (blue = included; grey = non included) then combined totals below in the bar chart (**B**). **A** In the “other” group in Abdelrazik et al. refers to exposed healthcare professionals (close contacts were a separate group in this trial too). For Cerutti et al., this refers to patients who were tested from “high risk” travel areas as deemed by the local government

The indication for testing for SARS-CoV-2 of the participants [e.g., screening or (a)symptomatic testing, close contacts] are included in Fig. 3, demonstrating that the systemic review contains a diverse population sample that would be representative of those being tested for COVID-19.

### Manufacturer of lateral flow device

**Fig. 4:**
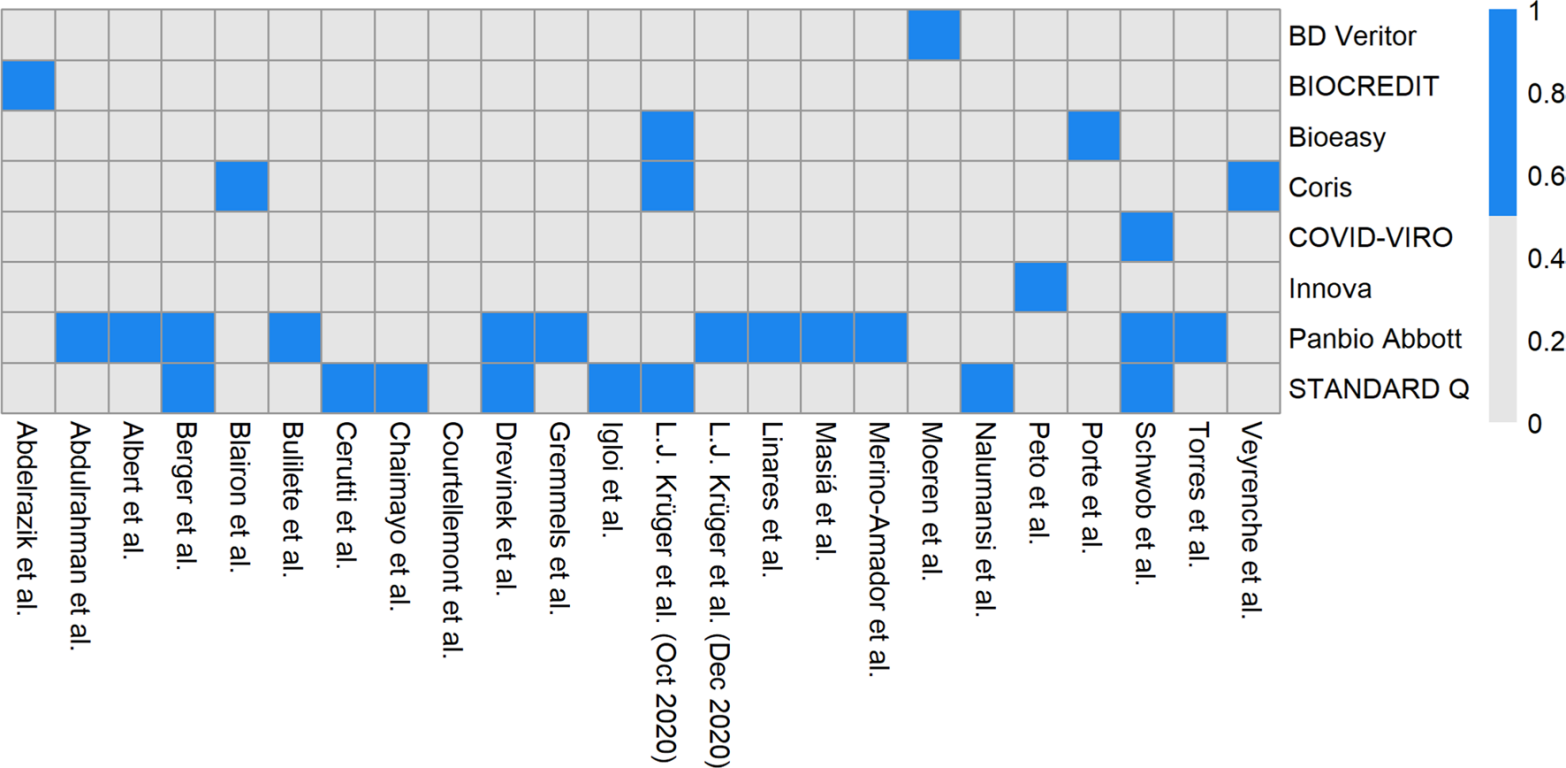
Heat map chart showing manufacturer of LFD test used in each individual paper. Blue = included; grey = not included

Eight different manufacturers of LFDs were used across 24 studies. Panbio Abbot had the highest number of publications and was used across 12 different studies with a combined total of 13,000 tests. This is demonstrated in Fig. 4 and Additional file 1: Appendix 2.

### Sensitivity and specificity data

**Fig. 5:**
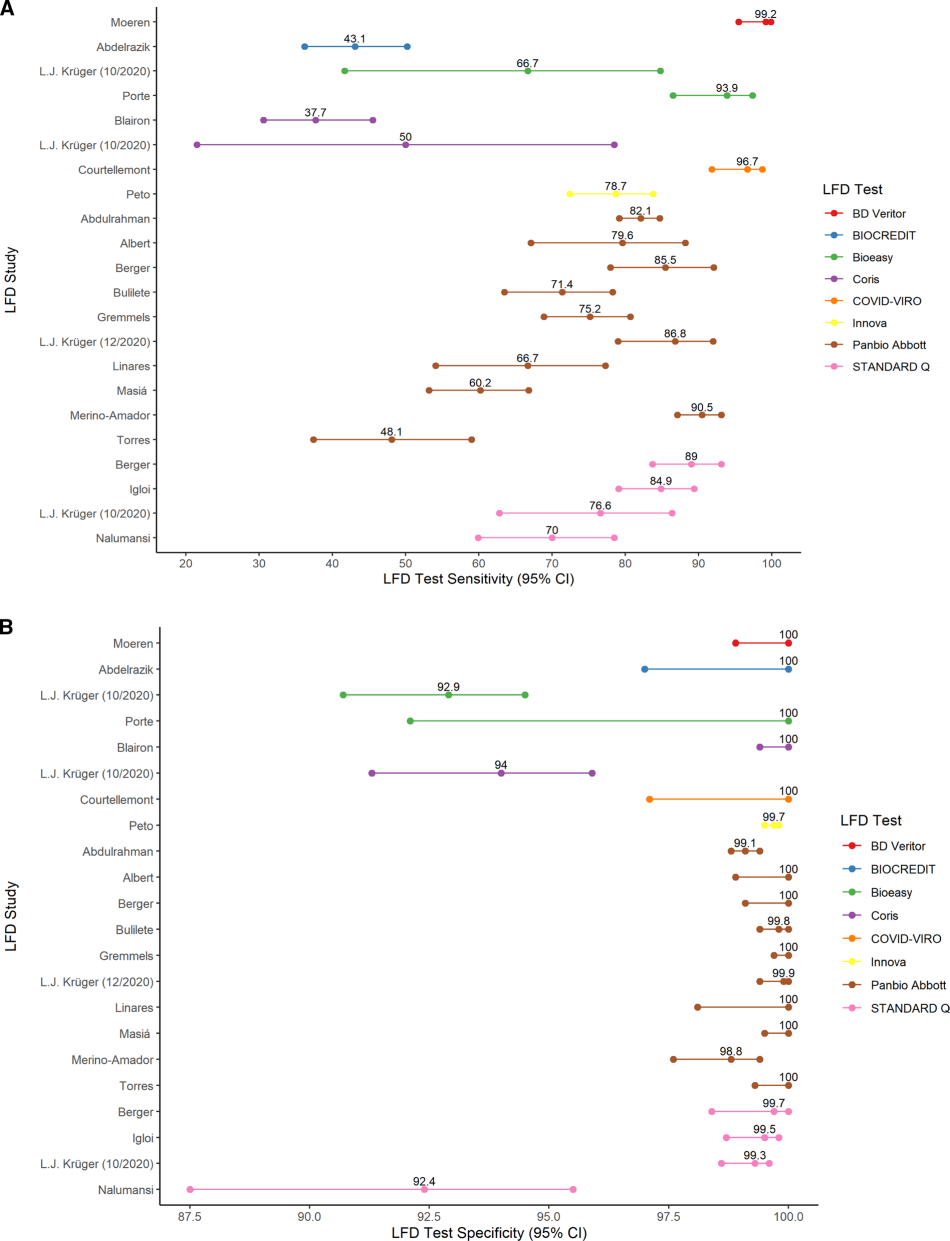
LFD sensitivity by study with 95% confidence intervals displayed in **A**. LFD specificity data by study with 95% confidence intervals displayed in **B**. Kruger et al. (2020) [25] tested three different types of LFDs hence three different results

Individual study sensitivity and specificity data is demonstrated by Table 2. This shows a range of sensitivity from 37.7% (95% CI 30.6–45.5) from Blairon et al. [16] (which used the CORIS LFD) to Moeren et al. [29] with a sensitivity of 99.2% (95% CI 95.5–99.9) using the BD Veritor LFD test, as demonstrated by Fig. 5A. For specificity, all studies demonstrated a specificity over 92%. Eleven studies had a specificity of 100%. This is demonstrated in Fig. 5B.

**Table 2:** Sensitivity and specificity data extracted from each study

Study	Sample size	True Positive	False Negative	False Positive	True Negative	Sensitivity	Sensitivity 95% CI Low	Sensitivity 95% CI High	Specificity	Specificity 95% CI Low	Specificity 95% CI High
Iglὁi et al. [23]	970	NA	NA	NA	NA	84.9	79.1	89.4	99.5	98.7	99.8
Berger et al. (Ag2) [15]	535	NA	NA	NA	NA	85.5	78.0	92.1	100.0	99.1	100.0
Berger et al. (Ag1) [15]	529	NA	NA	NA	NA	89.0	83.7	93.1	99.7	98.4	100.0
Abdelrazik et al. [12]	310	81	107	0	122	43.1	36.2	50.2	100.0	97.0	100.0
Abdulrahman et al. [13]	4183	602	131	30	3420	82.1	79.2	84.7	99.1	98.8	99.4
Albert et al. [14]	412	43	11	0	358	79.6	67.1	88.2	100.0	98.9	100.0
Blairon et al. [16] ^†^	774	60	99	0	615	37.7^†^	30.6^†^	45.5^†^	100.0	99.4	100.0
Bulilete et al. [17]*	1369	100	40	2	1220	71.4	63.5*	78.3*	99.8	99.4*	100.0
Chaimayo et al. [19]^†^	454^†^	64	-4	4	390	106.7^†^	NA^†^	NA^†^	99.0^†^	97.4^†^	99.6
Courtellemont et al. [20]	248	117	4	0	127	96.7	91.8	98.7	100.0	97.1	100.0
Drevinek et al. [21] (Ag1)	591	148	75	0	368	66.4	59.9	72.2	100.0	99.0	100.0
Drevinek et al. [21] (Ag2)*	591	141	82	2	366	63.2*	56.7	69.3	99.5	98.0	99.9
Gremmels et al. [22]^†^	1575	152	50	0	1373	75.2^†^	68.9^†^	80.7^†^	100.0	99.7	100.0
L.J. Krüger et al. [24] (2020)	1108	92	14	1	1001	86.8	79.0	92.0	99.9	99.4	100.0
L.J. Krüger et al. [25] (2020)	2417	50	20	85	2262	71.4	60.0	80.7	96.4	95.5	97.1
L.J. Krüger et al. [25] (2020) (Ag1)	1263	36	11	9	1207	76.6	62.8	86.4	99.3	98.6	99.6
L.J. Krüger et al. [25] (2020) (Ag2)	425	4	4	25	392	50.0	21.5	78.5	94.0	91.3	95.9
L.J. Krüger et al. [25] (2020) (Ag3)	729	10	5	51	663	66.7	41.7	84.8	92.9	90.7	94.5
Linares et al. [26]^†^	255	40	20	0	195	66.7^†^	54.1^†^	77.3^†^	100.0	98.1	100.0
Masiá et al. [27]*	913	118	78	0	709	60.2*	53.2	66.8	100.0	99.5	100.0
Merino-Amador et al. [28]	958	325	34	7	592	90.5	87.1	93.1	98.8	97.6	99.4
Moeren et al. [29]^†^	352	122	1	0	334	99.2^†^	95.5^†^	99.9^†^	100.0	98.9	100.0
Nalumansi et al. [30]	262	63	27	13	159	70.0	59.9	78.5	92.4	87.5	95.5
Peto et al. [31]	6954	155	42	22	6735	78.7	72.4	83.8	99.7	99.5	99.8
Porte et al. [32]	127	77	5	0	45	93.9	86.5	97.4	100.0	92.1	100.0
Torres et al. [34]	634	38	41	0	555	48.1	37.4	59.0	100.0	99.3	100.0
Veyrenche et al. [35]^†^	45†	13	32	0	0	28.9^†^	17.7^†^	43.4^†^	NA^†^	NA^†^	NA^†^
Schwob et al. [33]^†^	928	327	45	0	601	87.9^†^	84.2^†^	90.8^†^	100.0	99.4	100.0

*Shows data which had slight variations between our data calculations and the calculations made in the study, possibly due to a different method for calculating 95% confidence intervals.

^†^Shows data that produced significant differences in between our calculated data and the study’s data or it was not possible to calculate sensitivity and specificity from the data in the study

### Pooled data based on manufacturer of LFD

**Fig. 6:**
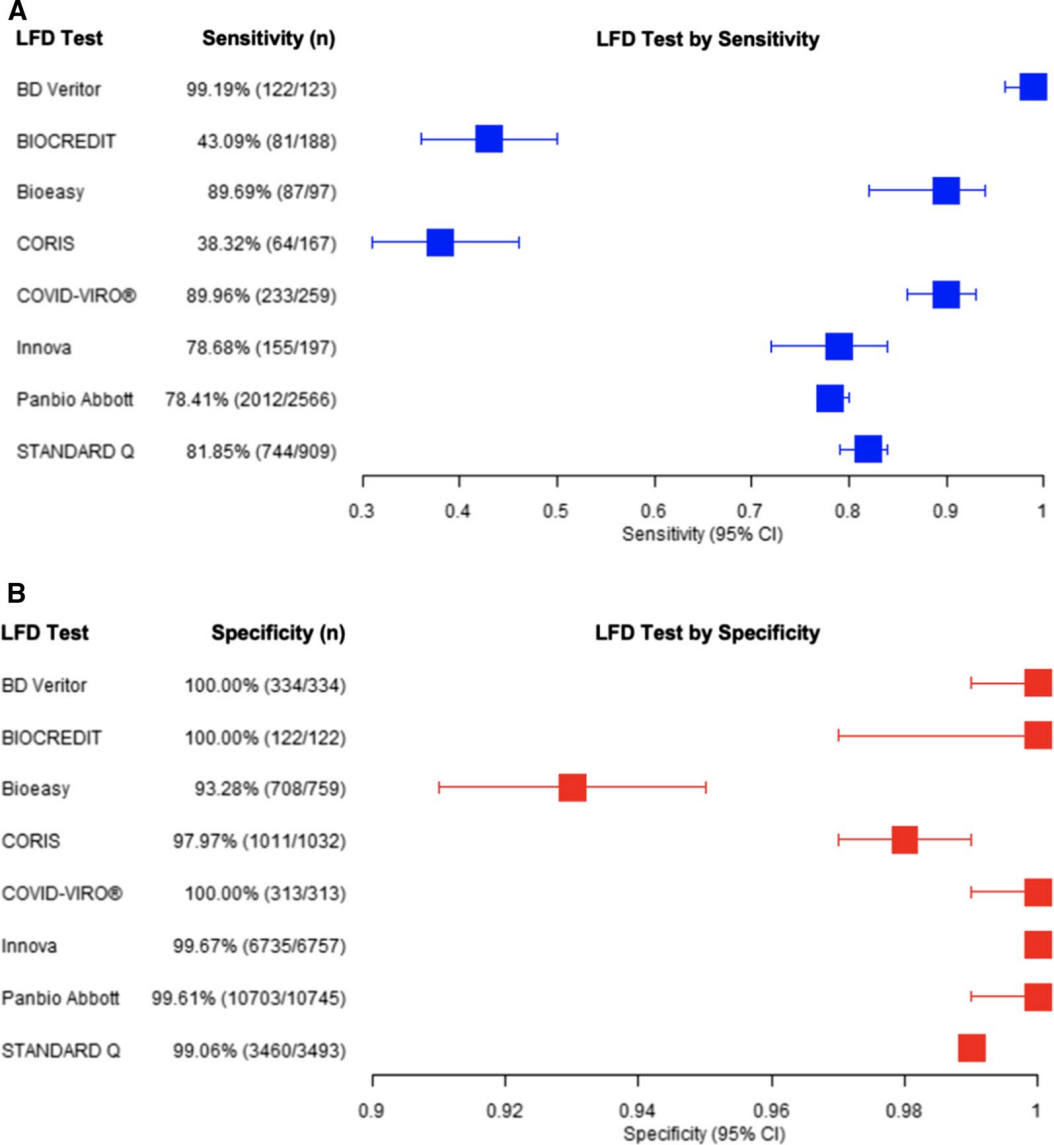
Pooled LFD sensitivity data based on manufacturer with 95% confidence intervals displayed in **A**. Pooled LFD specificity data based on manufacturer with 95% confidence intervals displayed in **B**

After combining studies based on manufacturer of LFD, BD Veritor had the best sensitivity of 99.19% (95% CI 95.54–99.86%), though the sample size was small and it was only tested from a single centre study. The CORIS and BIOSENSOR were the lowest sensitivity LFDs demonstrating sensitivities of less than 45%. Panbio Abbott has been most thoroughly evaluated and noted a sensitivity of 78.41% (95% CI 76.78–79.96%) across over 2500 individual tests. All manufacturers demonstrated a specificity of over 93% and three (BD Veritor, BIOCREDIT, COVID-VIRO) had specificities of 100%. This is shown in Table 3 and Fig. 6.

**Table 3:** Pooled sensitivity and specificity data based on manufacturer of LFD

Type of LFD test	Sample size	Positive sample size	LFD detected	Negative sample size	Number of negatives detected by LFD	Sensitivity %	Sensitivity 95% CI Low	Sensitivity 95% CI High	Specificity %	Specificity 95% CI Low	Specificity 95% CI High
Panbio Abbott	13,221	2566	2012	10,745	10,703	78.41	76.78%	79.96%	99.61	99.47%	99.71%
Innova	6954	197	155	6757	6735	78.68	72.44%	83.82%	99.67	99.51%	99.78%
STANDARD Q	4402	909	744	3493	3460	81.85	79.21%	84.22%	99.06	98.68%	99.33%
CORIS	1199	167	64	1032	1011	38.32	31.29%	45.88%	97.97	96.91%	98.67%
Bioeasy	856	97	87	759	708	89.69	82.05%	94.30%	93.28	91.27%	94.85%
COVID-VIRO®	572	259	233	313	313	89.96	85.70%	93.06%	100.00	98.79%	100.00%
BD Veritor	352	123	122	334	334	99.19	95.54%	99.86%	100.00	98.86%	100.00%
BIOCREDIT	310	188	81	122	122	43.09	36.21%	50.23%	100.00	96.95%	100.00%

### Sample collection comparison

**Fig. 7:**
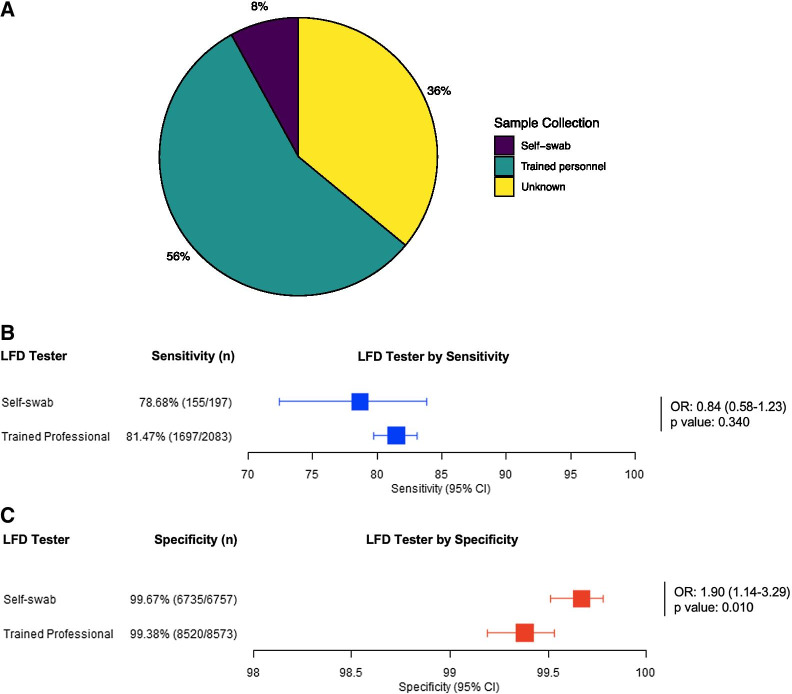
The proportions of LFD tests by sample collector is displayed in **A**. The sensitivity of LFD tests by sample collector with 95% confidence intervals is displayed as a Forest Plot in **B**. The specificity of LFD tests by sample collector with 95% confidence intervals is displayed as a Forest Plot in **C**

Studies were split by sample collector as displayed in Table 1. In fourteen studies the sample was collected by trained professionals; only the Peto et al. [31] study involved samples collected by the patient as part of self-swabbing, though with the test performed by a trained professional. Nine studies did not specify who the operator was. Trained professionals carried out 10,656 tests and 6954 were by self-swabbing as demonstrated in Fig. 7A. Sensitivity for trained professionals was 81.47% (95% CI 79.7–83.1) and for self-swabbing was 78.68% (95% CI 72.4–83.8) (see Fig. 7B, C). Both showed a specificity of over 99% as shown in Fig. 7C [trained professionals = 99.4% (95% CI 99.2–99.5); self-swabbing = 99.7% (95% CI 99.5–99.8)].

## Discussion

This systematic review has identified, across 24 studies and over 26,000 LFD tests, that a number of individual manufacturers of LFDs recorded a sensitivity of over 78% compared to the gold standard test of RT-PCR, with one individual manufacturer reaching up to 99.19% sensitivity in one single centred trial (BD Veritor). Specificity was more consistent, with over 92% in all individual studies and from the pooled data. The large variation between brands of LFDs could be due to several factors including individual study design, operator competencies but also quality of the LFD itself. This highlights the impressive performance of the Panbio Abbot and Innova brands both with sensitivities of over 78% but with a sample size of 13,221 and 6954 respectively.

This study is the first to summarise the existing body of studies to help create a broader understanding for LFD testing for SARS-CoV-2 and is the first systematic review of its kind. While RT-PCR is and is likely to remain the gold standard of testing, this study highlights the potential utility of rapid antigen testing to support RT-PCR in the scaling up of a country’s testing program to include mass testing, contact tracing programs and potentially surge-testing [9, 36]. Potential use of LFDs might be to provide short term additional capacity, or as an adjunct to PCR testing [1, 7, 8]. The lower sensitivity demonstrated by certain brands of LFDs compared to RT-PCR can be overcome to an extent in high prevalence areas with appropriate frequency of testing. LFDs may come into their own when used in areas with big spikes in cases. We note that there is an increasing body of modelling data highlighting that the best surveillance testing methods are tests that can be scaled up and reported quickly, [36] requirements which LFDs may have suitable characteristics. These models also highlight the need for recurrent testing. This again is a requirement LFDs can fulfil given their minimal expense. High frequency testing in high prevalence areas may negate some concerns around sensitivity [36]. In contrast, low incidence areas would expose the inferior sensitivities demonstrated by LFDs in this study, and RT-PCR would be the most suitable, especially if there is a reduction in demand for mass population and high frequency testing in these areas. This point highlights that whilst LFDs have some benefits, when compared directly to RT-PCR, their performance when detecting SARS-CoV-2 was inferior and as such they should be utilised when RT-PCR is overwhelmed.

Our study design is not without its limitations. There are possible confounding variables including the marked heterogeneity in terms of study designs whereby some targeted asymptomatic or symptomatic groups, and others targeted contacts of symptomatic patients. However, as there was a variety of settings and scenarios to replicate the conditions of real-life testing, this data can still provide valuable insight into the performance of LFDs.

Furthermore, this systematic review takes the assumption that for the diagnosis of COVID-19, RT-PCR testing is the most appropriate measure for comparison. There is a debate whether RT-PCR testing is the most appropriate method in a high-incidence setting [37]. In such a setting RT-PCR might actually report an overall greater number of positive cases than those which should be considered active infections, because of the presence of residual RNA which can be present for several months after an initial infection with SARS-CoV-2 [37–39]. Other measures of assessing the infectivity of individuals, such as viral culture, might provide better measurements but suffer from other logistical implementation issues.

On a final note, caution should be exerted particularly in view of new emergent strains. The sensitivity of any COVID-19 tests to new strains, not least LFDs must be confirmed. Several such evaluations have been completed by Public Health authorities in the United Kingdom and have given reassurance in this regards [40].

## Conclusions

In summary, this systematic review has shown that lateral flow devices can produce varying sensitivity and specificity results compared to the other forms of SARS-CoV-2 diagnostics. We have shown that a number of manufacturers of LFDs can produce high specificity but there is significant heterogeneity in sensitivity (38.32–99.19%), which may suit LFD use to high prevalence areas in an attempt to rapidly increase testing in areas with raised transmission. Our evidence gives support to the practice of self-swabbing for sample collection compared to the test being performed by a trained healthcare professional. LFDs potentially offer a new form of COVID-19 testing that might ease the pressure on the RT-PCR testing program. Enhanced capacity for mass testing, contact tracing and surge-testing, may in turn help stop the chain of transmission of COVID-19.

## Supplementary Information

**Additional file 1.** Appendix 1 - Gender split for each paper included in the study. Appendix 2 - Sample size based on manufacturer of LFD used.

## Data Availability

The datasets used and/or analysed during the current study are available from the corresponding author on reasonable request.

## Abbreviations

LFD: Lateral flow device
RT-PCR: Reverse transcriptase polymerase chain reaction
