# Supplementary material for: A systematic review of the sensitivity and specificity of lateral flow devices in the detection of SARS-CoV-2
Source: BMC Infect Dis. 2021 Aug 18;21:828. doi: 10.1186/s12879-021-06528-3 (PMC8371300; doi:10.1186/s12879-021-06528-3)
Supplement: Supplementary file 1 — Additional file 1. Appendix 1 - Gender split for each paper included in the study. Appendix 2 - Sample size based on manufacturer of LFD used. [file 12879_2021_6528_MOESM1_ESM.pdf]

Supplementary Materials

Appendix 1:

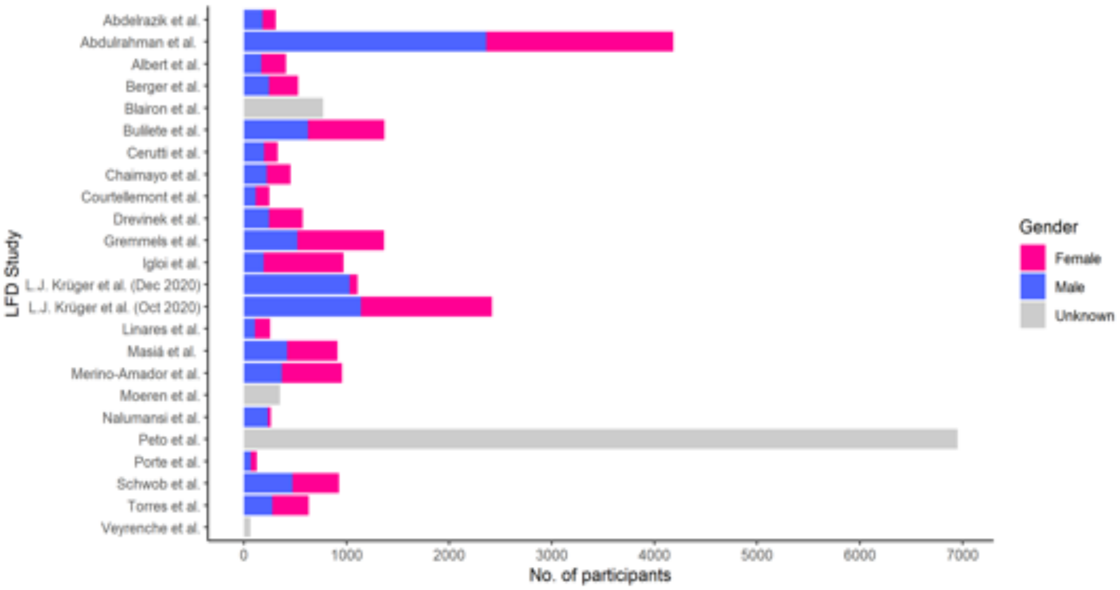

Gender split for each paper included in the study:

Appendix 2:

Sample size based on manufacturer of LFD used

**Manufacturer of LFD**	**Sample size**
Panbio Abbott	13221
Innova	6954
Standard Q	4402
CORIS	1199
Bioeasy	856
COVID-VIRO®	572
BD Veritor	352

BIOCREDIT	310
